# Improvement in Mung Bean Peptide on High-Fat Diet-Induced Insulin Resistance Mice Using Untargeted Serum Metabolomics

**DOI:** 10.3389/fnut.2022.893270

**Published:** 2022-04-29

**Authors:** Lina Li, Yu Tian, Yuchao Feng, Shu Zhang, Yingjun Jiang, Yiwei Zhang, Yuanyuan Zhan, Changyuan Wang

**Affiliations:** ^1^College of Food, Heilongjiang Bayi Agricultural University, Daqing, China; ^2^Library, Heilongjiang Bayi Agricultural University, Daqing, China

**Keywords:** mung bean peptides (MBPs), untargeted serum metabolomics, glucolipid metabolism, insulin-resistant (IR), obesity

## Abstract

This study aimed to elucidate the potential regulatory mechanism of mung bean peptides (MBPs) on glucolipid metabolism in insulin-resistant mice induced by high-fat diet (HFD) using untargeted serum metabolomics, enzyme linked immunosorbent assay (ELISA), intraperitoneal injection glucose tolerance test (IPGTT), insulin tolerance test (IPITT), and hematoxylin-eosin staining (H&E). The regulatory effect of MBPs for alleviating insulin resistance was studied by measuring body weight, fasting blood glucose (FBG) and serum insulin levels, C-Peptide levels, inflammatory and antioxidant factors, and histopathological observation of C57BL/6 mice. The experimental results showed that dietary intervention with MBPs (245 mg/kg/d) for 5 weeks significantly relieved insulin resistance in HFD mice. The body weight, insulin resistance index, and the levels of FBG, C-Peptide, IL-6, TNF-α, and MDA in the serum of HFD mice significantly decreased (*P* < 0.05). Conversely, SOD content and pancreatic β cell function index significantly increased (*P* < 0.05), and the damaged pancreatic tissue was repaired. One biomarker associated with insulin resistance was glycine. In addition, there were four important differential metabolites: pyroglutamate, D-glutamine, aminoadipic acid, and nicotinamide, involved in 12 metabolic pathway changes. It was found that MBPs may regulate amino acid, glycerol phospholipid, fatty acid, alkaloid, and nicotinamide metabolism to regulate the metabolic profile of HFD mice in a beneficial direction.

## Introduction

According to a 3-year study on obesity published in The Lancet in 2019, the global obesity rate has been increasing annually and has become the most prominent global chronic disease that endangers human health (1). One-third of the world's population is overweight, and obesity rates have increased by 50% in adults and doubled in children and adolescents in the last 20 years. According to the Report on Nutrition and Chronic Diseases in China (2020), over 50% of Chinese adults were overweight and obese in 2020, making China one of the major obese countries in the world. Studies have found that obesity can cause symptoms of hyperglycaemia and insulin resistance in the body, and the combination of the two can cause impaired glucose tolerance. Obesity also increases the body's inflammatory response and oxidative stress response, increasing the degree of insulin resistance (2). Kanasaki have proved that obesity, induced by an HFD, is usually accompanied by systemic insulin resistance (IR) (3). The HFD-fed mouse model is considered a reliable model for studying obesity, and related metabolic complications (4). Existing studies have shown that IR is closely related to obesity, type 2 diabetes mellitus (T2DM), non-alcoholic fatty liver disease, and other metabolic disorders (5). Long-term drug use produces side effects on the human body, such as gastrointestinal and liver diseases. Therefore, it is of great significance to find a safe, efficient, and small side effect of natural biologically active substance adjunctive drugs or alternative drugs to intervene in glucose and lipid metabolism disorders.

In recent years, many studies have shown that supplementation with foodborne functional components can effectively improve glucose and lipid metabolism disorders (6). Existing studies have shown that dietary peptides can promote weight loss and hypoglycaemia (7). The polypeptide Vglycin extracted and isolated by Jiang et al. from soybean or pea seeds and the tetrapeptide isolated by Asokan et al. from soybean protein hydrolysates can effectively reduce the bodyweight of mice and improve IR (3, 8). Hashidume et al. extracted and separated the hormone-like peptide insulin from green soybean (Echigomidori). Kwak et al. obtained a peptide from black beans for hypoglycaemic experiments *in vivo*, both of which significantly reduced the blood glucose of mice, indicating that legume peptide has good hypoglycaemic activity (9, 10).

Mung beans (*Vigna radiata* L.) are a traditional Chinese medicine food homology grain or legume, high in nutritional value and balanced composition (11). It also has a lot of potential health benefits and, like Qing Rejiedu, reduces bodily heat, improve oxidation, lower blood sugar, reduce lipids in the blood, and has immune regulating functions (12), and study the polyphenols and polysaccharides and peptides are the main functional component (13). It has been reported that mung beans have abnormal sugar and lipid metabolism. For example, Hou et al. found that dietary supplementation of cooked mung beans or mung bean powder can effectively reduce the bodyweight of mice (14, 15). Nakatani et al. found that mung bean protein (MPI) rich in 8S globulin also reduced weight gain induced by a high-fat diet. Kohno et al. in Japan found that GLUCODIA^TM^ could inhibit fasting blood glucose and insulin levels (16, 17). These studies showed that the consumption of whole mung beans or mung bean protein had certain effects on weight loss and glucose reduction, but the specific active ingredients and mechanism of action were not clear. Mung bean protein intake in the body, through digestive enzymes, is hydrolysed into peptides. As a microactive bioactive ingredient, peptides are likely to be the main components of mung bean and mung bean protein. However, there are few reports on the hypoglycaemic activity of MBPs.

Glucose and lipid metabolism disorders can cause metabolic changes in the body. Metabolomics is a new technology for studying glycolipid metabolism by analyzing abnormal metabolic changes caused by diseases and screening out differential metabolites and metabolic pathways using high-throughput detection and data analysis. The use of metabolomics to study glucose and lipid metabolism disorders may lead to early intervention through biomarkers or reduce the risk of disease and complications (18). In this study, the effect of MBPs on regulating glucose and lipid metabolism disorders was evaluated based on physiological and biochemical indicators, histopathological observations, and untargeted serum metabolomics. Principal component analysis (PCA) and orthogonal partial least squares discriminant analysis (OPLS-DA) were used for cluster analysis to screen differential metabolites and explore the regulatory role of MBPs in the glucolipid metabolism pathway. The results of this study can provide theoretical support for the design of hypoglycaemic and lipid-lowering functional foods.

## Materials and Methods

### Materials and Reagents

Specific pathogen-free (SPF) C57BL/6 male mice were purchased from Liaoning Changsheng Biotechnology Co., Ltd., with the production license number SCXK (Liao) 2015–0001. Standard and high-fat feed were purchased from Nantong Trophy Feed Technology Co., Ltd. Mung bean protein powder was purchased from Shandong Zhaoyuan Wenji Food Co., Ltd. Insulin (ML001983-J), C-Peptide (ML063022-J), IL-6 (ML063159-J), TNF-α (ML002095-J), SOD (ML643059-J), and MDA (ML826369-j) detection kits were obtained from Shanghai Enzyme-linked Biotechnology Co., Ltd. A blood glucose meter was purchased from Roche (Shanghai, China). All other reagents were analytically pure.

### MBPs Preparation

The mung bean protein powder (protein mass fraction 80%) was prepared in a 30 L solution with a 10% substrate mass fraction. The pH value was adjusted to 8.0 with 4 mol/L NaOH solution, and 2.0% (substrate mass) alkaline protease (2.4 Au/g) was added and stirred in a water bath at 55°C. The pH value was kept constant with 4 mol/L NaOH solution, the enzymatic hydrolysis was terminated for 5 h, the pH value was adjusted to 7.0, the enzyme was inactivated at 100°C for 10 min while being stirred, and the mixture of MBPs was obtained. After filtration and desalting, spray drying was performed. After the outlet temperature of spray drying was 180°C and the inlet temperature was 80°C. The prepared MBPs powder was refrigerated for later use (19).

### Inhibitory Effect of MBPs on α-Glucoside

All sample solutions were prepared from phosphate buffer (0.1 M) at a pH of 6.9. The final concentration of α-glucoside was 0.5 U/mL, the concentration of p-nitrobenzene α-d-glucopyranoside (pPNG) was 2 mM, and that of MBPs was 0.5–7 mg/mL. A 50 μL α-glucosidase solution was mixed with a 50 μL MBPs solution and set at 37°C for 10 min. The 50 μL of pPNG solution was then added to the mixed solution to start the reaction. After mixing, the samples were incubated at 37°C for 20 min. Finally, 100 μL of sodium carbonate solution (1 M) was added to the reaction solution to stop the reaction, and the absorbance was measured at 405 nm. Formula 1-1 was used to calculate the α-glucosidase inhibition rate of MBPs samples:

α-Glucosidase inhibition rate (%) = [1-(A_sample_-A_control−1_)/A_control−2_] × 100 (1-1),

where, A_sample_ is the absorbance value of the mixture of MBPs, enzyme, and pPNG, A_control−1_ is the absorbance value of the mixture after the buffer solution replaces the enzyme solution, and A_control−2_ is the absorbance value of the buffer solution instead of the sample solution (18).

### Physicochemical Properties of MBPs

The test was performed according to GB5009.3-2016 first method, GB5009.4-2016 first method, GB5009.5-2016 first method, and GB5009.6-2016 second method.

### Animal Treatment

**Figure 1:**
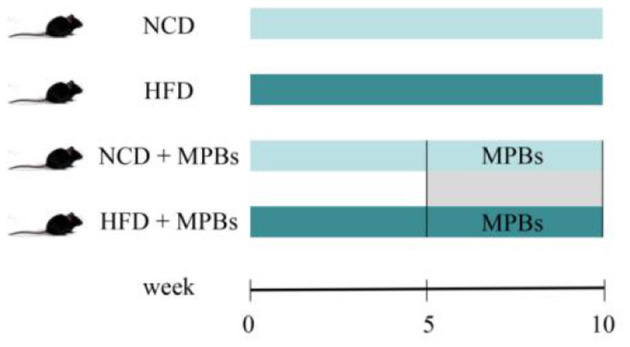
Animal experiment design.

Forty-six-week-old C57BL/6 male mice, weighing 18–20 g, were reared under SPF conditions with light and dark cycles for 12 h, room temperature of 25 ± 3°C, and relative humidity of 50 ± 15%. Animal experiments were performed according to the animal experiment guidelines of the Heilongjiang Bayi Agricultural University Experimental Center. After 1 week of adaptive feeding, the mice were randomly divided into two groups (*n* = 20): the NCD (normal diet) and HFD (high-fat diet) groups. After feeding for 5 weeks, the mice were weighed, the FBG level was detected, and the glucose tolerance test was performed by intraperitoneal injection to verify the success of the model. After successful modeling, the groups were divided into the NCD (normal diet), NCD + MBPs (normal diet + MBPs), HFD (high-fat diet), and HFD + MBPs (high-fat diet + MBPs) groups for the dietary intervention experiment [*n* = 10; (5)]. MBPs were added to drinking water, and dietary supplements were taken freely, with the added concentration of 245 mg/kg (20). The body weight of the mice was recorded weekly, and the feed intake and MBPs water consumption were recorded daily. Experimental groups are shown in Figure 1.

### FBG, IPGTT, and IPITT Measurement

After modeling, FBG level was checked once a week, after 2 weeks of MBPs dietary intervention. During blood collection, a small incision was made at the end of the rat's tail with a surgical blade to squeeze out the amount of blood required for the subject. After disinfection with iodophor, the rat was placed back into the cage and was monitored to prevent infection or tail severing. At the end of the modeling and 1 week before MBPs dietary intervention, the mice underwent overnight fasting (12 h) and were intraperitoneally injected with 2 g/kg body weight glucose solution at the end of the experiment. Blood samples were obtained from the tail vein before and 15, 30, 60, and 120 min after treatment to detect blood glucose levels. At the end of the MBPs dietary intervention experiment, the mice underwent fasting for 3 h and then received an intraperitoneal injection of insulin at a dose of 0.75 U/kg body weight. Blood samples were collected from the tail vein before and 15, 30, 60, 75, and 90 min after injection to detect blood glucose levels. The feed and water were supplemented immediately after the experiment. Blood glucose values at each time point were connected with curves, and the area under the curve (AUC) was the AUC value (4).

### Serum Biochemical Indicator Measurement

The levels of insulin, C-Peptide, IL-6, TNF-α, SOD, and MDA in the serum were determined by ELISA. The specific methods are described in the instructions.

### Histopathological Examination

The pancreas tissues of the mice were taken and fixed in 4% (v/v) paraformaldehyde/PBS fixative solution, buried in paraffin wax, and then cut into 5 μm slices. These slices were dyed with H&E staining, and tissue morphology was observed using an optical microscope (Olympus, Tokyo, Japan) (×200) (3).

### Untargeted Serum Metabolomic Analysis

#### Metabolites Extraction

A 20 μL sample was transferred into an EP tube, added with 80 μL extract (methanol: acetonitrile = 1:1) (v/v), including isotope-labeled standard internal mixture, after vortex mixing for 30 s and ice water bath ultrasound for 10 min, and was allowed to stand at −40°C for 1 h. The sample was centrifuged at 12,000 rpm (centrifugal force, 13,800 × g; radius, 8.6 cm) at 4°C for 15 min, and the supernatant was placed in a sample bottle for on-machine testing. All samples were mixed with the same amount of supernatant to prepare QC samples for on-machine testing (4).

#### LC-MS/MS Analysis

The target compounds were separated using a Waters ACQUITY UPLC BEH Amide (2.1 mm × 100 mm, 1.7 μm) liquid chromatography column. Phase A was aqueous, containing 25 mmol/L ammonium acetate and 25 mmol/L ammonia water, and phase B was acetonitrile. The sample tray temperature was 4°C, and the injection volume was 3 μL. Thermo Q Exactive HFX Mass Spectrometer using Xcalibur Thermo control software and primary and secondary mass spectrometry data acquisition. Specific parameters were set as follows: sheath gas flow rate, 30 Arb; Aux gas flow rate, 25 Arb; capillary temperature, 350°C; and full MS resolution, 60,000. The MS/MS resolution was 7,500. The collision energy was 10/30/60 in the NCE mode. Spray voltage was 3.6 kV (positive) or −3.2 kV (negative).

#### Data Pre-processing and Annotation

ProteoWizard software was used to convert the original data into mzXML format. The programme package, independently written as XCMSR, was used for peak recognition, peak extraction, peak alignment, and integration and was then matched with the BiotreeDB (V2.1) self-built secondary mass spectrometry database for material annotation. The cut-off value of the algorithm was set at 0.3.

### Statistical Analysis

SPSS19.0 software was used for the analysis of variance (ANOVA) to compare the significant differences among the groups. All results are expressed as mean ± standard deviation, *P* < 0.01, and *P* < 0.05 and were significantly different at different levels. ^*^P < 0.05, ^**^P < 0.01, NCD compared with HFD mice. ^#^*P* < 0.05, ^##^*P* < 0.01, HFD compared with HFD + MBPs mice.

## Results

### Preparation and Physical-Chemical Properties of MBPs

As shown in Table 1, the degree of hydrolysis was 26.42% after 5 h of enzymatic hydrolysis using alkaline protease (2.4 Au/g). The MBPs hydrolysate was filtered with four layers of 300 wood yarns, and impurities such as unhydrolysed protein were removed. The solid matter content in the feed solution was 8.0–8.3%. The conductivity was 8.30 and 2.70 ms/cm before and after desalting, respectively. The powder was prepared by spray drying, and the physicochemical properties of the prepared MBPs powder were tested, and the results are shown in Table 1. The inhibitory rate of α-GLA was 42.17 ± 2.23%, indicating that the MBPs prepared by this method have a certain hypoglycaemic effect.

**Table 1:** Expanded experiment of the preparation of MBPs and its physical and chemical properties.

**Degree of hydrolysis (%)**	**Moisture (g/100 g)**	**Ash (g/100 g)**	**Fat (g/100 g)**	**Protein (g/100 g)**	**Peptide (g/L)**	**α-GLA inhibitory rate (%)**
26.42	3.5 ± 0.028	3.0 ± 0.28	0.9 ± 0	79 ± 0.57	3.67 ± 0.22	42.17 ± 2.23

### Effects of MBPs on IR Mice's Body Weight, Food Intake, and Water Intake

**Figure 2:**
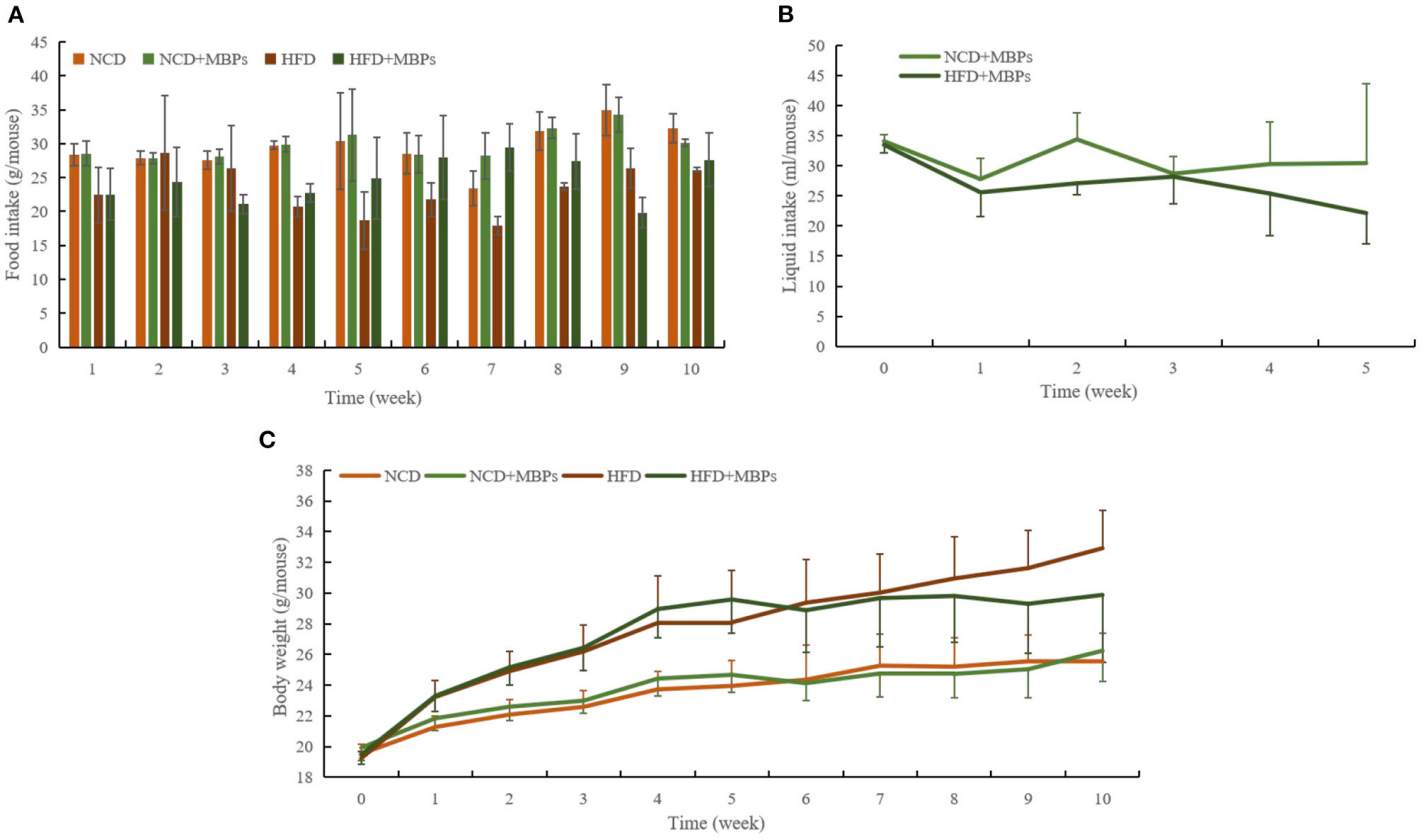
Effects of MBPs on **(A)** food intake, **(B)** water intake, and **(C)** body weight in different mice groups. Data were presented as the mean ± SD (*n* = 10).

During modeling, the intake of an HFD was lower than that of an ordinary diet (Figure 2A), but the HFD group showed significant obesity and fat accumulation. As shown in Figure 2C, at the end of modeling, the weight of mice in the NCD group was 23.948 ± 1.636 g, and that of the HFD group was 28.833 ± 2.805 g. The importance of mice in the HFD group was 20.40% overweight compared to that of the NCD group, which met the standard of obesity model modeling. Throughout the MBPs dietary intervention, there were no significant changes in food intake and body weight in mice in the NCD and NCD + MBPs groups (Figures 2A,C). One week after MBPs dietary intervention, compared with the HFD group, the bodyweight of mice in the HFD + MBPs group was decreased by 1.67% (*P* < 0.05). At the late stage of MBPs dietary intervention, the food intake of the HFD + MBPs group was higher than that of the HFD group. However, the weight gain was significantly lower than that of the HFD group (*P* < 0.05). After dietary intervention with MBPs, the bodyweight of HFD mice decreased by 9.24%, indicating that dietary supplementation with MBPs can effectively inhibit weight gain and obesity caused by HFD.

As shown in Figure 2B, before the MBPs intervention, the drinking water volume of mice fed with normal diet and HFD was 34.1 ± 1.1 mL and 33.5 ± 1.4 mL, respectively. After the MBPs intervention, the drinking water volume of mice fed with normal diet and HFD was 27.8 ± 3.4 mL and 25.6 ± 4.0 mL, respectively, significantly reduced (*P* < 0.05). The dietary intervention amount of MBPs was adjusted according to the weekly water intake to ensure consistent nutritional information of MBPs in the two groups of mice. The final dietary intervention amount of MBPs was 244.69 and 246.18 mg/kg, respectively, in the NCD + MBPs and HFD + MBPs groups.

### Effects of MBPs on IR Mice: FBG, IPGTT, and IPITT

**Figure 3:**
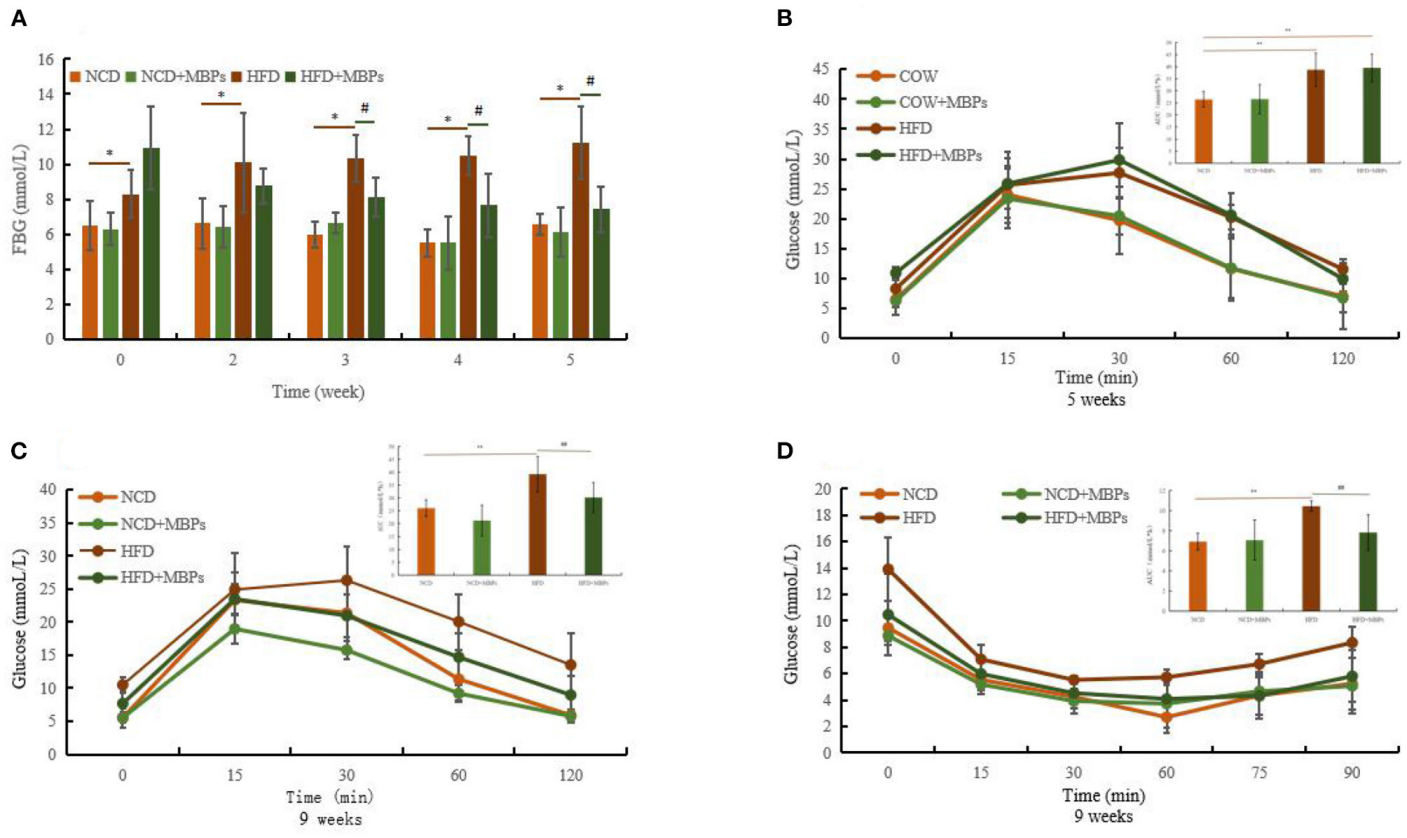
Effects of MBPs on **(A)** FBG, **(B)** IPGTT (5 weeks) and AUC, **(C)** IPGTT (9 weeks) and AUC, **(D)** IPITT (9 weeks) and AUC. Data were presented as the mean ± SD (*n* = 10). *P < 0.05, **P < 0.01, NCD compared with HFD and HFD + MBPs mice. ^#^*P* < 0.05, ^##^*P* < 0.01, HFD compared with HFD + MBPs mice.

As shown in Figure 3A, at the end of modeling, the FBG of the NCD group was 6.41 ± 1.16 mmol/L, and that of the HFD group was 9.62 ± 1.87 mmol/L, indicating a significant difference between the two groups (*P* < 0.05). In the whole process of MBPs dietary intervention, compared with the NCD group, the FBG of the mice in the NCD + MBPs group was slightly decreased, but the difference was not significant.

After 1 week of MBPs dietary intervention, FBG levels decreased in the HFD + MBPs group compared with the HFD group, but the difference was insignificant. Three weeks after MBPs dietary intervention, there was a significant difference in blood glucose levels between the two groups (*P* < 0.05). After 5 weeks of MBPs dietary intervention, FBG levels in the HFD + MBPs group were 24.93% lower than those in the HFD group. These results suggest that MBPs may play a beneficial role in stabilizing the level of blood glucose in the early stages of dietary intervention.

Glucose tolerance was measured using an IPGTT. After the mice were fed with HFD diet for 5 weeks, the peak of the blood glucose level of the mice in the HFD and NCD groups appeared at 30 and 15 min, respectively (Figure 3B). The area under the glucose tolerance curve is usually used to evaluate the degree of glucose tolerance (Figure 3B). Compared with the NCD group, the HFD group mice showed glucose intolerance combined with the body weight of mice. This proves that the IR model was successfully constructed. After 4 weeks of MBPs dietary intervention, the peak of the blood glucose level appeared at 30 min in the HFD-fed mice, and the peak of the blood glucose level occurred at 15 min in the HFD + MBPs group as in those fed with a normal diet. Compared with the HFD group, the blood glucose level of the HFD + MBPs group could be restored to a relatively normal level more quickly, and glucose tolerance significantly improved (Figure 3C). These results indicate that dietary intervention with MBPs can effectively improve glucose tolerance in insulin-resistant mice.

Insulin tolerance was measured using an IPITT. After 5 weeks of MBPs dietary intervention, the lowest blood glucose level of the HFD group was 30 min, and that of the other groups was 60 min. The lowest blood glucose level of the NCD + MBPs and HFD + MBPs groups was higher than that in the NCD group (Figure 3D). The mice in the HFD + MBPs group had a more significant ability to clear blood glucose than those in the HFD group, suggesting that dietary intervention with MBPs can effectively improve insulin tolerance, blood glucose level, and islet function in insulin-resistant mice. The results showed that dietary supplementation with MBPs alleviated HFD-induced abnormal glucose tolerance and IR, thus improving glucose homeostasis in mice.

### Effects of MBPs on IR Mice: Serum Insulin and C-Peptide Levels

**Figure 4:**
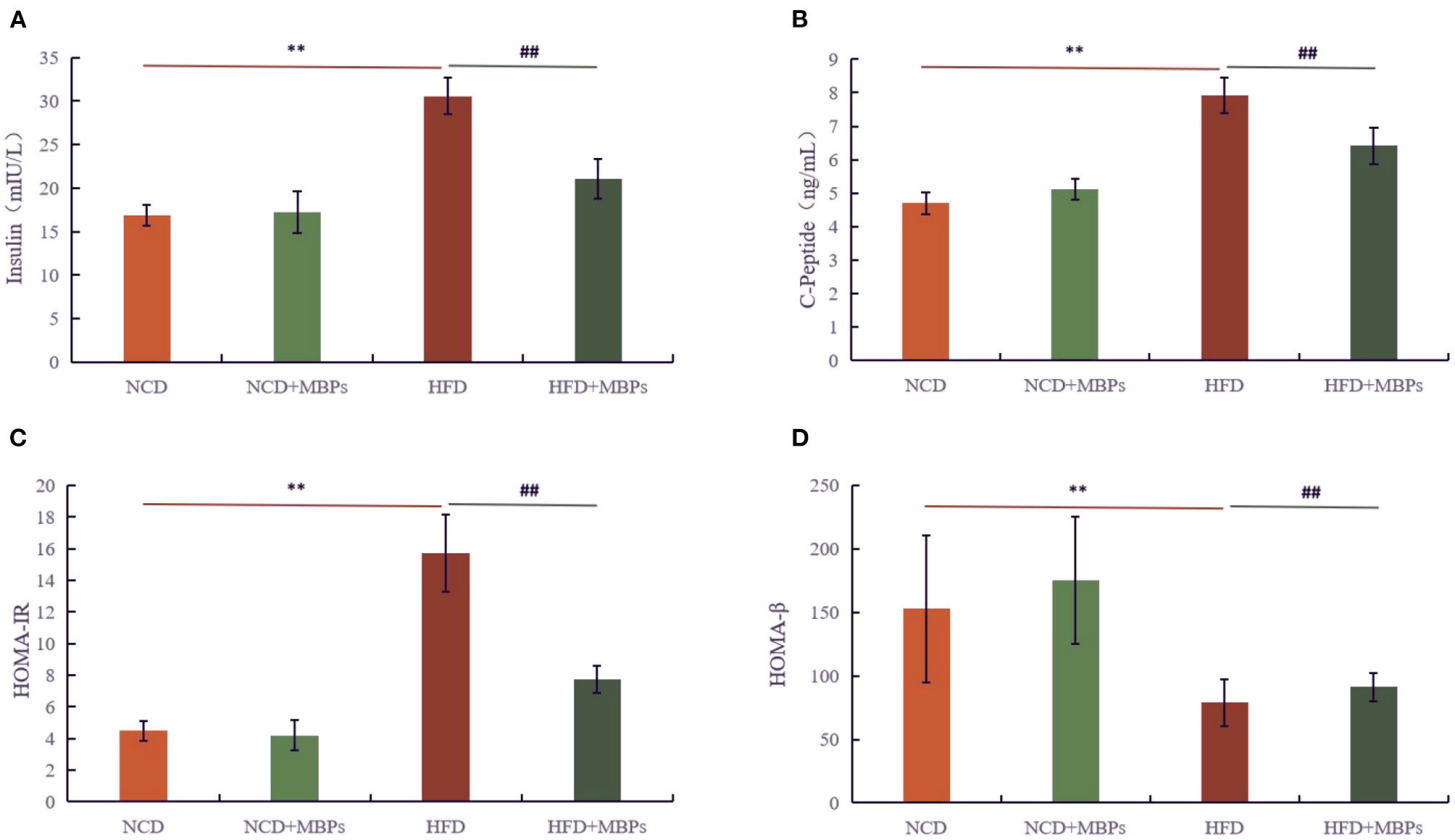
Effects of MBPs on **(A)** insulin levels, **(B)** C-Peptide levels, **(C)** HOMA-IR, and **(D)** HOMA-β. Data were presented as the mean ± SD (*n* = 6). **P < 0.01, NCD compared with HFD mice. ^##^*P* < 0.01, HFD compared with HFD + MBPs mice.

The function of islet β cells was determined by detecting the levels of insulin and C-Peptide in the serum of mice, and the improvement effect of MBPs on IR in mice was evaluated. Figures 4A,B show that after 5 weeks of dietary intervention, compared with the NCD group, the serum insulin and C-Peptide levels in the NCD + MBPs group did not differ significantly, while the serum insulin and C-Peptide levels in the HFD group increased significantly (*P* < 0.01). Compared with the HFD group, the levels of insulin and C-Peptide in the serum of mice in the HFD + MBPs group significantly decreased (*P* < 0.01). The results showed that the function of the islets of mice improved after dietary intervention with MBPs.

IR and islet β cell function was evaluated by calculating the IR (HOMA-IR) and islet β cell function (HOMA-β) indices (Figures 4C,D). After 5 weeks of dietary intervention, there was no significant difference in the HOMA-IR value between the NCD and NCD + MBPs groups, but HOMA-β was significantly different (*P* < 0.05). Compared with the NCD group, the HOMA-IR value in the HFD group significantly increased (*P* < 0.01), and the HOMA-β value significantly decreased (*P* < 0.01). Compared with the HFD group, the HOMA-IR value in the HFD + MBPs group significantly decreased (*P* < 0.01), and the HOMA-β value significantly increased (*P* < 0.05). This suggests that dietary intervention with MBPs can effectively improve the islet function in insulin-resistant mice.

### Effects of MBPs on IR Mice: Serum Inflammatory Factor Levels

**Figure 5:**
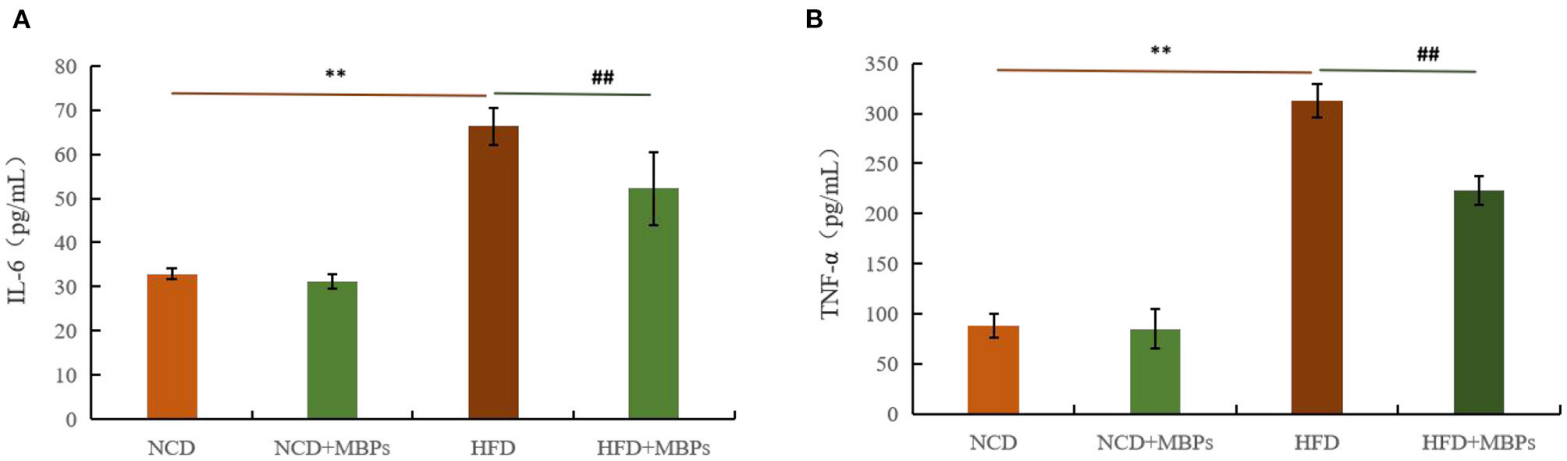
Effects of MBPs on **(A)** IL-6 levels and **(B)** TNF-α levels. Data were presented as the mean ± SD (*n* = 6). **P < 0.01, NCD compared with HFD mice. ^##^*P* < 0.01, HFD compared with HFD + MBPs mice.

To evaluate the anti-inflammatory effect of MBPs in insulin-resistant mice, serum IL-6 and TNF-α levels were measured. Figures 5A,B show that after 5 weeks of MBPs dietary intervention, compared with the NCD group, the serum levels of IL-6 and TNF-α in the NCD + MBPs group were not significantly different, while those in the HFD group were significantly increased (*P* < 0.01). These results indicate that the HFD diet can increase the serum levels of pro-inflammatory factors in mice and induce systemic inflammation. Compared with the HFD group, the serum IL-6 and TNF-α levels in the HFD + MBPs group were decreased by 21.18 and 28.56%, respectively (*P* < 0.01). The results showed that the inflammatory environment was improved, indicating that MBPs dietary intervention can effectively improve systemic inflammation in mice.

### Effects of MBPs on IR Mice: Serum Antioxidant Factor Levels

**Figure 6:**
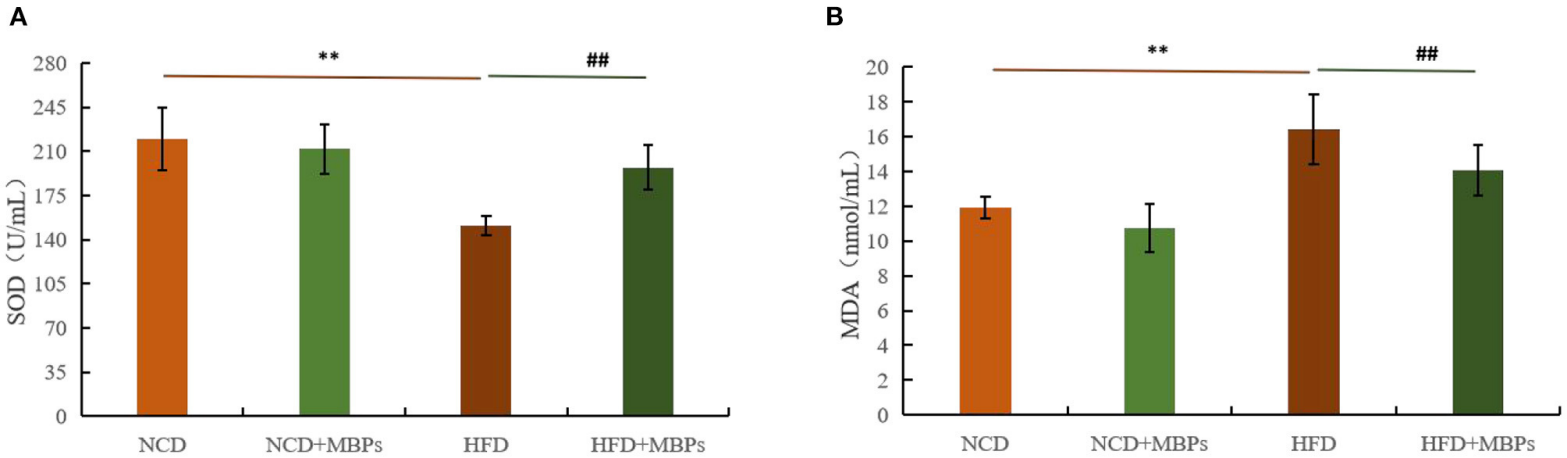
Effects of MBPs on **(A)** SOD levels and **(B)** MDA levels. Data were presented as the mean ± SD (*n* = 6). **P < 0.01, NCD compared with HFD mice. ^##^*P* < 0.01, HFD compared with HFD + MBPs mice.

The antioxidant effect of MBPs in insulin-resistant mice was evaluated by measuring the levels of SOD and MDA in the serum in mice. Figures 6A,B show that after 5 weeks of MBPs dietary intervention, compared with the NCD group, the serum levels of SOD in the NCD + MBPs group were not significantly different, while MDA content was significantly decreased (*P* < 0.05). The serum levels of SOD in the HFD group were significantly decreased (*P* < 0.01), while the MDA content was significantly increased (*P* < 0.05). These results indicated that HFD induced an oxidative stress response in mice. Compared with the HFD group, the serum levels of SOD in the HFD + MBPs group increased by 30.50% (*P* < 0.01), and MDA content decreased by 14.39% (*P* < 0.01). The results showed that MBPs had an excellent antioxidant effect, and dietary intervention with MBPs could effectively improve the oxidative stress response in mice.

### Effect of MBPs on IR Mice: Pancreatic Histopathology

**Figure 7:**
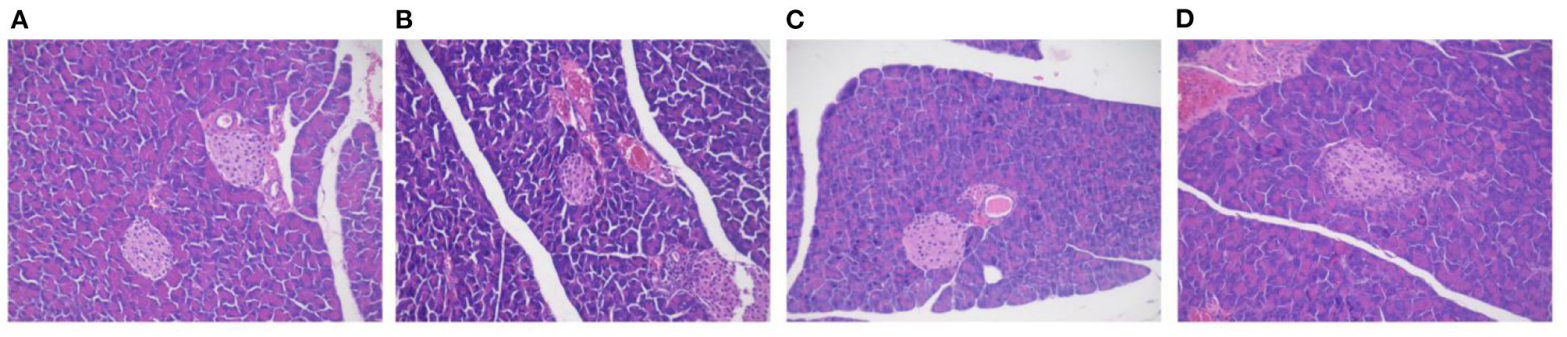
Effects of MBPs on histopathological changes on pancreas stained with H&E [**(A)** NCD, **(B)** NCD + MBPs, **(C)** HFD, **(D)** HFD + MBPs]. Magnification, 200 X.

The pancreatic acinar tissues in mice in each group were arranged neatly and closely. Adenosine cells were conical, and acinar cells were rich in proenzyme particles (Figure 7). The overall structure of the pancreatic tissue of mice in the NCD and NCD + MBPs groups was standard, with the islet visible and regular arrangement of islet cells and without obvious degeneration of islet cells, such as proliferation, degranulation, and cavitation. In the HFD group, the overall structure of the pancreatic tissue was abnormal, islet atrophy was apparent, and the number of islet cells decreased sharply. The general design of the pancreatic tissue in the HFD + MBPs group was relatively standard. H&E staining showed that MBPs could improve pancreatic tissue damage and restore islet cell morphology.

### Untargeted Serum Metabolic Analysis

These results indicate that MBPs dietary intervention can improve insulin resistance in HFD-fed mice. To further explore the positive impacts of MBPs on IR through the metabolic pathway by UHPC-QE HFX-MS.

### Multivariate Statistical Analysis Using PCA and OPLS-DA

**Figure 8:**
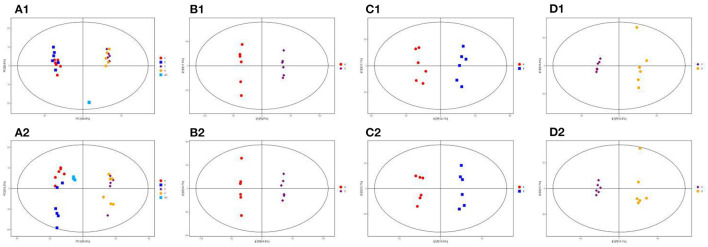
PCA score plots of the QC, NCD, NCD + MBPs, HFD, and HFD + MBPs groups in **(A**_**1**_**)** NEG and **(A**_**2**_**)** POS ion modes; OPLS-DA analysis of serum of mice. Score scatter plot of OPLS-DA model for the group NCD vs. HFD [**(B**_**1**_**)** NEG ion, **(B**_**2**_**)** POS ion], NCD vs. NCD + MBPs [**(C**_**1**_**)** NEG ion, **(C**_**2**_**)** POS ion], HFD vs. HFD + MBPs [**(D**_**1**_**)** NEG ion, **(D**_**2**_**)** POS ion].

PCA analysis was performed to understand the metabolic changes in the NCD, NCD + MBPs, HFD, and HFD + MBPs (Figures 8A_1_, A_2_). The results show that all samples are in the 95% confidence interval (Hotelling's T-squared ellipse), and the quality control models (blue dots) are closely clustered together, indicating that the instrument stability is good reliable data. In the NEG and POS ion modes, 12 samples from the NCD and NCD + MBPs groups, and 12 samples from the HFD and HFD + MBPs groups were located on both sides, it indicated that the HFD diet changed the metabolites of the mice.

Owing to the complex multidimensional characteristics of metabolic data, simple PCA model analysis could not distinguish the differences between samples. Therefore, OPLS-DA was used to further analyse the data. The OPLS-DA score diagram showed that the NCD and HFD groups were significantly separated under the two modes of the metabolic curve (Figures 8B_1_, B_2_). This shows that the analytical model is successfully established, and the model has good predictive ability. The two ion modes in NCD with NCD + MBPs groups (Figures 8C_1_, C_2_) and HFD with HFD + MBPs groups (Figures 8D_1_, D_2_) were also well-separated, the results showed that MBPs had certain effect on serum metabolites of mice. The PCA and OPLS-DA results further confirmed the successful establishment of the HFD-induced IR mouse model, and MBPs dietary intervention could regulate the metabolic status of mice.

### Hierarchical Clustering Analysis

**Figure 9:**
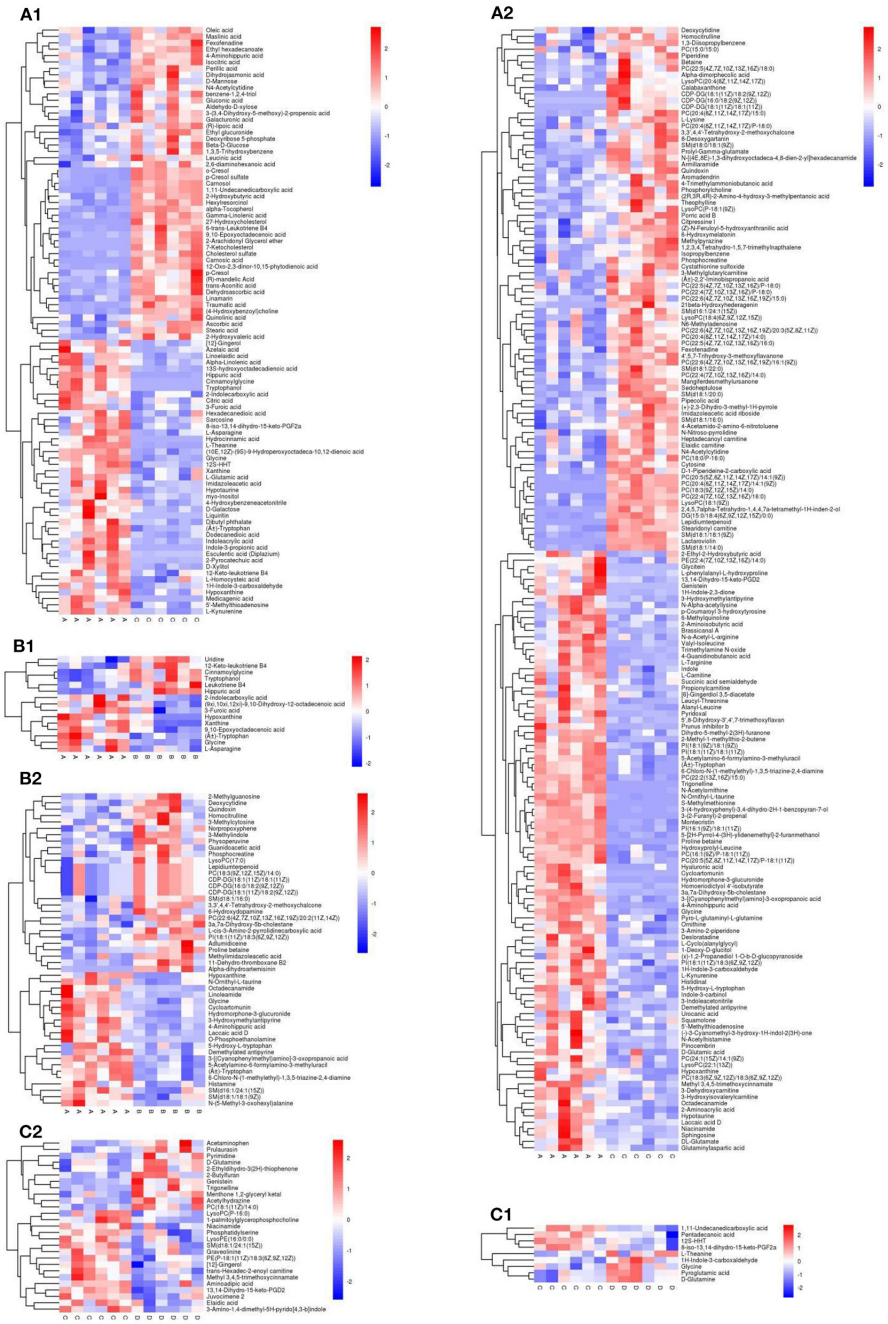
Heat map for the group NCD vs. HFD [**(A**_**1**_**)** NEG ion, **(A**_**2**_**)** POS ion], NCD vs. NCD + MBPs [**(B**_**1**_**)** NEG ion, **(B**_**2**_**)** POS ion], HFD vs. HFD + MBPs [**(C**_**1**_**)** NEG ion, **(C**_**2**_**)** POS ion]. Metabolites that were significantly up-regulated were red, metabolites that were significantly down-regulated were blue, and metabolites that were not significantly different were gray.

The thresholds of VIP > 1 and *P* <0.05, were used to screen the differential metabolites. Using cluster analysis, 258 different metabolites were identified in the NCD and HFD groups, with 92 and 176 metabolites in NEG and POS modes, respectively, and 10 metabolites were duplicated (Figures 9A_1_, A_2_). The differential metabolites were mainly divided into 10 categories: lipids and lipoid-like molecules (89), organ heterocyclic compounds (48), organic acids and derivatives (45), organic nitrogen compounds (24), benzenoids (19), phenylpropanoids and polyketides (15), nucleosides, nucleotides, and analogs (5), alkaloids and derivatives (2), organooxygen compounds (1), organosulfur compounds (1), and other compounds (9). A total of 132 differential metabolites were upregulated, and the compounds were mainly organ heterocyclic compounds (31), organic acids and derivatives (31), and lipids and functions-like molecules (30). A total of 126 differential metabolites were down-regulated, and the compounds were mainly lipids and functions-like molecules (59), organ heterocyclic compounds (17), organic acids and liposomes were mainly derived from derivatives (14), organic nitrogen compounds (13), etc.

A total of 61 differential metabolites were identified in the NCD and NCD + MBPs groups, with 15 and 49 metabolites in NEG and POS modes, respectively, and three metabolite was duplicated (Figures 9B_1_, B_2_). The differential metabolites were mainly divided into seven categories: organic acids and derivatives (28), lipids and lipoid-like molecules (18), benzenoids (5), nucleosides, nucleotides, and analogs (3), phenylpropanoids and polyketides (3), alkaloids and derivatives (2), and other compounds (2). Fifteen differential metabolites were down-regulated, and the compounds were mainly organic acids and derivatives (5), organ heterocyclic compounds (4), etc. A total of 27 differential metabolites were upregulated, and the compounds were mainly organic acids and derivatives (17), lipids and lipid-like molecules (5), and benzenoids (3). Thirty-four differential metabolites were down-regulated, and the compounds were mainly Lipids and lipid-like molecules (13), and organic acids and derivatives (11), etc.

A total of 35 differential metabolites were identified in the HFD and HFD + MBPs groups, with nine and 27 metabolites in NEG and POS modes, respectively, and one metabolite was duplicated (Figures 9C_1_, C_2_). The differential metabolites were mainly divided into eight categories: lipids and lipoid-like molecules (14), organ heterocyclic compounds (7), organic acids and derivatives (6), benzenoids (3), phenylpropanoids and polyketides (2), alkaloids and derivatives (1), organooxygen compounds (1), and other compounds (1). Twenty differential metabolites were upregulated, and the compounds were mainly lipids and lipid-like molecules (13), organ heterocyclic compounds (3), and benzenoids (2). Fifteen differential metabolites were down-regulated, and the compounds were mainly organic acids and derivatives (5), organ heterocyclic compounds (4), etc.

We can learn that HFD-diet can cause the changes of organoheterocyclic compounds, organic acids and derivatives, lipids and functions-like metabolites from the results of the variation of the different metabolites. We found that changes in organoheterocyclic compounds metabolites were unique to HFD mice after MBPs dietary intervention, but no such changes were observed in NCD mice. From the perspective of differential metabolite changes, lipids and lipid-like molecules, organ heterocyclic compounds, organic acids and derivatives, and benzenoids are key substances in the IR intervention of HFD mice, and the pathway involved may play an important regulatory role.

To further investigate specific key differential metabolites, the thresholds of VIP >2 and *P* < 0.05 were used to further screen for differential metabolites (Table 2). In NEG and POS mode, a total of four key differential metabolites were screened in the NCD group compared with the HFD group, among which the content increased by two and decreased by two. A total of 19 key differential metabolites were screened in the NCD group compared with the NCD + MBPs group, among which the content increased by 14 and decreased by five. A total of 17 key differential metabolites were screened in the HFD group compared with the HFD + MBPs group, among which the content increased by seven and decreased by ten. Compared with the NCD group, the serum glycine content in the HFD group was significantly decreased (*P* < 0.01); compared with the HFD group, serum glycine content in the HFD + MBPs group was significantly increased (*P* < 0.05). Therefore, glycine is very likely to be a biomarker of MBPs to alleviate insulin resistance, which is closely related to hyperglycaemia (21), and a marker metabolite for diabetic nephropathy (22). Glycine is also significantly associated with obesity (23), non-alcoholic fatty liver (24), and liver injury (25).

**Table 2:** Detection of differential significant metabolites in serum samples.

**Metabolites**	**RT (s)**	**m/z**	**NCD vs. HFD**			**NCD vs. NCD** **+** **MBPs**			**HFD vs. HFD** **+** **MBPs**		
			**Fold**	**Change VIP**	**Trend**	**Fold**	**Change VIP**	**Trend**	**Fold**	**Change VIP**	**Trend**
Glycine	377.644	74.02401062	2.112531076	1.823370373	↑**				0.848350026	1.860017309	↓*
Acetylhydrazine	304.84	75.05595456							0.727000997	2.293493673	↓*
Pyrimidine	51.77945	81.04527191							0.79421204	2.027054377	↓*
3-(2-Furanyl)-2-propenal	25.56375	123.0442772	6123.067924	2.002923609	↑**						
2-Butylfuran	32.4606	125.0963537							0.606313313	2.185998749	↓*
L-Asparagine	397.1435	131.0457497				1.443001982	2.023292021	↑*			
2-Ethyldihydro-3(2H)-thiophenone	387.858	131.0534261							0.883659055	2.067508731	↓*
3-Methylindole	33.76045	132.0809081				0.428522942	2.137649557	↓**			
Hypoxanthine	177.4535	135.0307459				5.044082712	2.262354512	↑**			
Trigonelline	298.0175	138.0550135							0.432444636	2.545394309	↓*
Proline betaine	284.482	144.1019934	47.78931462	2.002965544	↑***						
D-Glutamine	387.825	147.076462							0.891753075	2.025599544	↓*
Xanthine	228.113	151.0258535				3.857517453	2.164399344	↑**			
Acetaminophen	333.112	152.070847							0.631311814	2.150588984	↓*
Tryptophanol	207.105	160.0765093				0.519125053	2.115966193	↓**			
Aminoadipic acid	463.326	162.0762193							1.221215364	2.010431339	↑*
Hippuric acid	212.2135	178.0506607				0.471955001	2.626718353	↓**			
4-Aminohippuric acid	305.4175	184.0906893				2.695651709	2.310264009	↑**			
6-Chloro-N-(1-methylethyl)-1,3,5-triazine-2,4-diamine	275.8305	188.0707864				1.295803631	2.171864595	↑**			
N-(5-Methyl-3-oxohexyl)alanine	191.699	202.1437956				1.817869266	2.079203554	↑*			
3-Hydroxymethylantipyrine	75.6506	205.0970828				7.591157165	2.147713611	↑**			
(Â±)-Tryptophan	275.8305	205.0972628				1.329512922	2.317694094	↑***			
3-[(Cyanophenylmethyl)amino]-3-oxopropanoic acid	221.361	219.0764398				1.5548966	2.430364577	↑***			
4-Acetylamino-6-formylamino-3-methyluracil	275.821	227.0791119				1.854534999	2.318067104	↑***			
1,11-Undecanedicarboxylic acid	278.637	243.1604145							1.348132392	2.629833431	↑***
Genistein	24.4093	271.0598034							0.247372353	2.472222275	↓*
Graveolinine	175.8815	280.0965224							1.613900184	2.117391715	↑*
Octadecanamide	114.862	284.294365				2.34503347	2.177107831	↑*			
Alpha-dihydroartemisinin	413.615	285.1667894				0.012369385	2.107925189	↓*			
9,10-Epoxyoctadecenoic acid	93.1316	295.2280172				1.449396345	2.282900413	↑**			
Menthone 1,2-glyceryl ketal	453.206	298.1007049							0.691277693	2.101830361	↓*
Juvocimene 2	184.874	299.2001634							1.84123869	2.115595941	↑*
Laccaic acid D	322.37	315.0519826				2.088903144	2.022309342	↑*			
12-Keto-leukotriene B4	91.99785	333.2073924				0.722304981	2.008487495	↓*			
13,14-Dihydro-15-keto-PGD2	183.744	353.2317097							1.873231024	2.17892745	↑**
trans-Hexadec-2-enoyl carnitine	197.505	398.3262737							1.455100418	2.356462472	↑**
Lepidiumterpenoid	97.1651	405.3723219	0.002648552	2.001760141	↓***						
Hydromorphone-3-glucuronide	373.7065	462.180626				2.290451384	2.129253122	↑**			
1-palmitoylglycerophosphocholine	214.535	496.3392124							1.163911585	2.076500405	↑*
PC(18:3(9Z,12Z,15Z)/14:0)	170.113	728.5216321	0.000714337	2.001727357	↓***						
PC(18:1(11Z)/14:0)	80.8706	732.5528628							0.700613567	2.213139087	↓*

↑ indicates increasing in content. ↓ indicates reducing in content. *P < 0.05, **P < 0.01, ***P < 0.001.

### Metabolic Pathway Analysis

**Figure 10:**
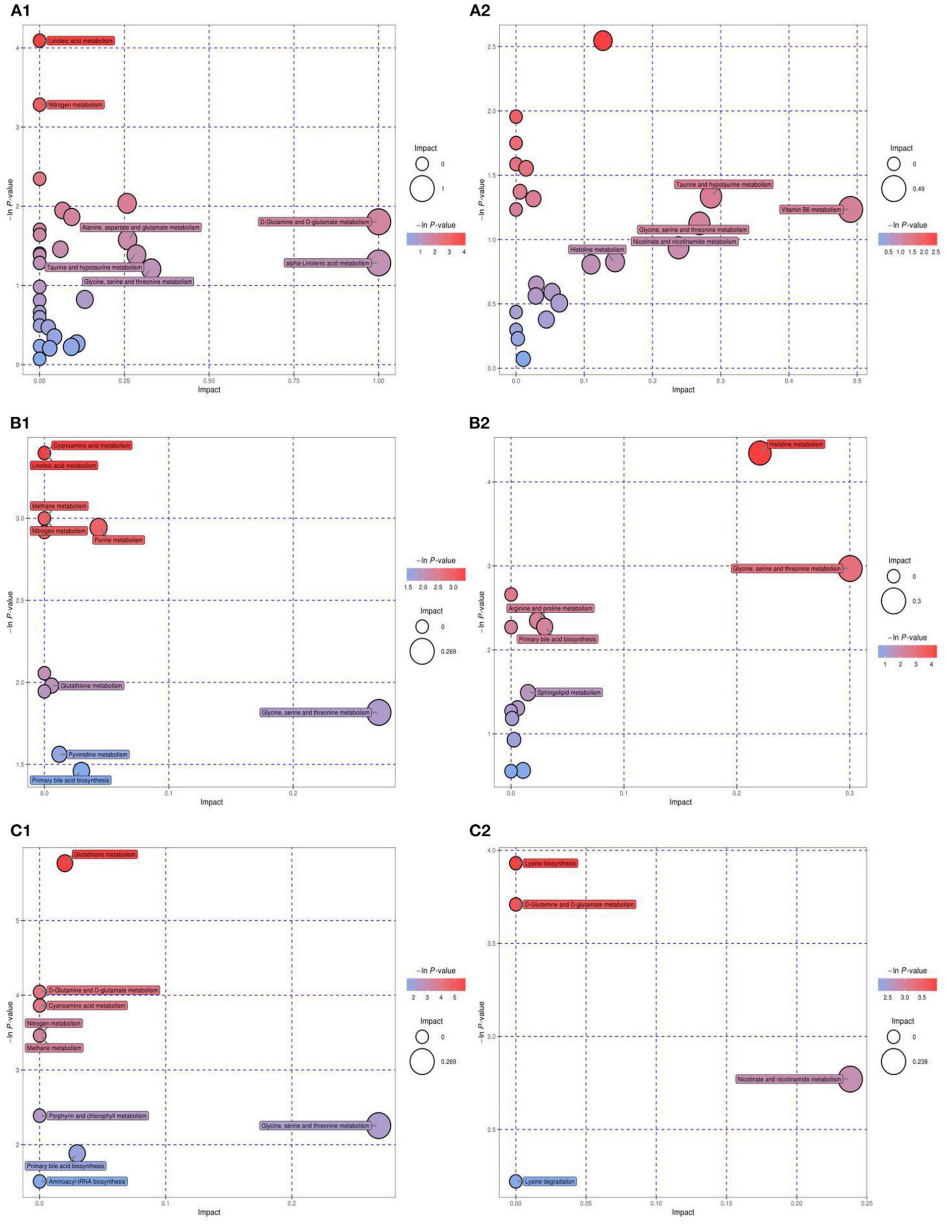
KEGG pathway analysis and differential abundance analysis diagram. NCD vs. HFD [**(A**_**1**_**)** NEG ion, **(A**_**2**_**)** POS ion], NCD vs. NCD + MBPs [**(B**_**1**_**)** NEG ion, **(B**_**2**_**)** POS ion], HFD vs. HFD + MBPs [**(C**_**1**_**)** NEG ion, **(C**_**2**_**)** POS ion]. Different colors of pathways indicate that pathways belong to different metabolic classifications. Line segments indicate the up-regulated or down-regulated of this pathway. Positive value of line segments indicates the overall up-regulation of this pathway; otherwise, it indicates the overall down-regulation of this pathway. The size of the end point of the line segment indicates the amount of substance annotated in the pathway.

Metabolic pathway enrichment analysis was conducted using KEGG data to explore the most relevant pathways and possible mechanisms of IR mitigation after dietary intervention with MBPs (Figure 10).

Compared with the NCD group, HFD-diet resulted in changes in 38 metabolic pathways (Figures 10A_1_, A_2_), involving 43 metabolites. Glycine, an important metabolite, is involved in aminoacyl-tRNA biosynthesis, cyanoamino acid metabolism, glutathione metabolism, glycine-serine-threonine metabolism, methane metabolism, nitrogen metabolism, porphyrin and chlorophyll metabolism, primary bile acid bio synthesis, and etc. After MBPs dietary intervention, compared with the NCD group, 16 metabolic pathways were changed in the NCD + MBPs group (Figures 10B_1_, B_2_), involving 13 metabolites. Compared with the HFD group, 12 metabolic pathways were changed in the HFD + MBPs group (Figures 10C_1_, C_2_), involving five metabolites. It was found that glycine metabolites in both NCD + MBPs and HFD + MBPs groups were involved in the changes of the above eight metabolic pathways. These results further indicated that glycine plays an important regulatory role in the two metabolic processes of insulin resistance induced by HFD-diet and insulin resistance alleviated by MBPs.

The HFD + MBPs group had four distinct metabolic pathways compared to the NCD + MBPs group, they are D-glutamine and D-glutamate metabolism, lysine biosynthesis, lysine degradation, and nicotinate-nicotinamide metabolism. These four pathways may be the unique mechanism by which MBPs alleviates insulin resistance induced by HFD-diet in mice.

To further understand the effects of MBPs on metabolic pathways in insulin resistant mice induced by HFD diet, we analyzed the differences in metabolic pathways and metabolites between HFD group with HFD +MBPs group (Table 3). It was found that pyroglutamic acid, D-glutamine, adipic acid, and nicotinamide were also involved in the regulation of multiple metabolic pathways, we speculated that they were important differential metabolites in the regulation of insulin resistance by MBPs, while glycine was an important biomarker in this process.

**Table 3:** Identified differential metabolites between the HFD group and HFD + MBPs group.

**Pathway**	**Total**	**Hits**	**Raw p**	**–ln(p)**	**Holm adjust**	**FDR**	**Impact**	**Hits Cpd**
Glutathione metabolism	26	2	0.003131	5.7664	0.25674	0.25674	0.02004	Glycine cpd:C00037; pyroglutamic acid cpd:C01879
D-Glutamine and D-glutamate metabolism	5	1	0.017543	4.0431	1	0.51496	0	D-Glutamine cpd:C00819
Cyanoamino acid metabolism	6	1	0.021022	3.8622	1	0.51496	0	Glycine cpd:C00037
Methane metabolism	9	1	0.0314	3.4609	1	0.51496	0	Glycine cpd:C00037
Nitrogen metabolism	9	1	0.0314	3.4609	1	0.51496	0	Glycine cpd:C00037
Porphyrin and chlorophyll metabolism	27	1	0.091834	2.3878	1	1	0	Glycine cpd:C00037
Glycine, serine and threonine metabolism	31	1	0.10485	2.2553	1	1	0.26884	Glycine cpd:C00037
Primary bile acid biosynthesis	46	1	0.15231	1.8818	1	1	0.02976	Glycine cpd:C00037
Aminoacyl-tRNA biosynthesis	69	1	0.22117	1.5088	1	1	0	Glycine cpd:C00037
Lysine biosynthesis	4	1	0.019635	3.9305	1	1	0	Aminoadipic acid cpd:C00956
Nicotinate and nicotinamide metabolism	13	1	0.062608	2.7709	1	1	0.2381	Niacinamide cpd:C00153
Lysine degradation	23	1	0.10845	2.2214	1	1	0	Aminoadipic acid cpd:C00956

## Discussion

### MBPs Alleviate Insulin Resistance in HFD Mice

In this study, obese mice were selected as the animal model because obesity is a typical metabolic disorder, which can lead to insulin resistance and blood glucose disorder. In severe cases, it can also develop into T2DM. While appropriating intervention on insulin resistance caused by obesity can effectively avoid the formation of T2DM.Dietary intervention is a mild and effective way of regulation. Sun et al. (6) and others found that corn peptide has the function of reducing blood glucose and repairing pancreatic injury (pancreas is closely related to the regulation of blood glucose stability), indicating that plant-derived peptide has the potential of T2DM prevention. After the MBPs dietary intervention, the levels of FBG, FINS, and C-Peptide significantly reduced (*P* < 0.05), HOMA-IR significantly decreased (*P* < 0.05), HOMA-β significantly increased (*P* < 0.05), and the damaged pancreatic tissues in HFD mice were repaired. The results showed that insulin resistance was reduced, and blood glucose stability was improved in HFD mice. These results are the same as those of sun, indicating that MBPs also has good hypoglycemic activity.

More and more scholars believe that obesity is a kind of systemic inflammation, which is closely related to the occurrence and development of insulin resistance and glucose and lipid metabolism disorders (26). After the MBPs dietary intervention, the levels of pro-inflammatory factors IL-6 and TNF-α in the serum of HFD mice significantly decreased (*P* < 0.05), SOD and MDA levels of serum antioxidant factors in HFD mice significantly improved (*P* < 0.05). These results indicated that systemic inflammation and oxidative stress were controlled in HFD mice. Sun et al. found that the hypoglycemic mechanism of corn peptide is to inhibit the phosphorylation of pro-inflammatory protein p38 and activate the phosphorylation of anti-apoptotic protein Akt (ser473) by reducing the expression of pro-inflammatory cytokine IL-6 (6). Mojibian et al. found that wheat peptide can reduce the contents of pro-inflammatory cytokines TNF-αand IL-6 in peripheral blood monocytes of patients with type 1 diabetes mellitus (T1D), and it plays a role in controlling T1D (27). Xie et al. found that mung bean peptide (MBPHS-I) with a molecular weight < 3 kDa had the best chelating activities on DPPH, hydroxyl radical, superoxide anion radical, and Fe^2+^ (28). Ngoh and Gan isolated heptapeptide sequences from pinto bean protein and showed significant anti-diabetic and antioxidant activities (29). The results of this study are consistent with existing research results, indicating that MBPs dietary intervention can comprehensively control blood glucose, body weight, and insulin resistance by reducing the content of pro-inflammatory factors and improving the antioxidant capacity of the body.

### Regulation of MBPs and Serum Metabolism in HFD Mice

**Figure 11:**
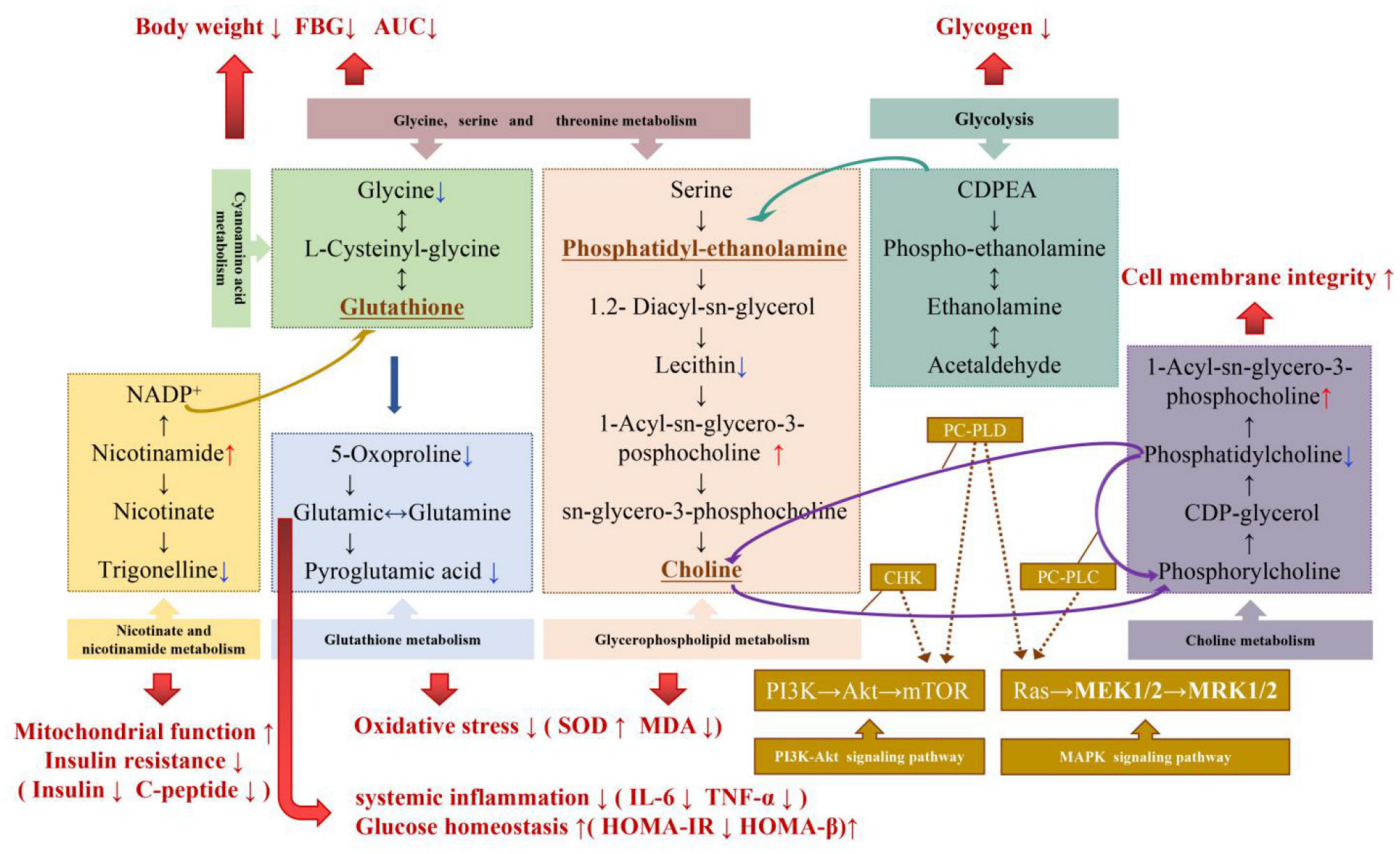
Potential biomarker and metabolic pathways involved in the regulation of IR by MBPs. Metabolites that were significantly up-regulated were red, metabolites that were significantly down-regulated were blue.

Based on the analysis and induction of different metabolites and metabolic pathways, it was found that MBPs may regulate the metabolic profile of HFD mice in a beneficial direction by regulating amino acid metabolism, glycerol phospholipid metabolism, lipid metabolism, alkaloid metabolism, and niacin and nicotinamide metabolism. Figure 11 illustrates the relationship between these pathways and the changes in related metabolites under the influence of MBPs.

The results showed that the regulation of amino acid metabolism might be the manifestation or potential mechanism of MBPs alleviating HFD-induced insulin resistance. Glycine is an essential product of glycine-serine-threonine metabolism, and generates glutathione (GSH) through cyanuric acid metabolism (30). GSH is an essential substance for scavenging ROS in the body, participating in the tricarboxylic acid cycle, and sugar metabolism, promoting the metabolism of sugars, fats, and proteins. GSH is metabolized to produce glutamate, which is recycled by the gamma-glutamyl cycle to produce glutamine. Glutamine can be converted from glucose in the body to produce the energy molecule, adenosine triphosphate (ATP), which acts as an energy source for the brain and effectively inhibits the body's craving for sugar. It has been proven that improving glutamine metabolism can regulate IR (31), glutamate/glutamine is positively correlated with obesity and IR (32). In addition, glutamine can maintain intestinal permeability in patients with severe pancreatitis and inhibit the release of inflammatory mediators. Simultaneously, glutamate can be further converted to pyroglutamate. Pyroglutamate improves glucose tolerance, and reducing pyroglutamate may improve glucose levels (33). Aminoadipic acid is a metabolic intermediate of the lysine synthesis pathway; lysine and phenylalanine have been confirmed to strongly stimulate insulin secretion (34). By regulating lysine biosynthesis and degradation, insulin secretion level can be improved, insulin resistance index can be reduced, islet β cell function index can be increased, and insulin resistance can be alleviated. These results are similar to those of the present study. Dietary intervention with MBPs can stabilize blood glucose and reduce body weight by regulating glycine-serine-threonine metabolism and cyanuric acid metabolism. By regulating glutamine metabolism, improving insulin secretion level, minimizing IR index, improving pancreatic β cell function index, and reducing systemic inflammation, by regulating glutathione metabolism, the oxidative stress response of the body can be improved. Metabolomics analysis suggested that the regulation of amino acid metabolism might be the manifestation or potential mechanism, by which, MBPs alleviates HFD-induced IR.

Dysregulation of glycerol phospholipid metabolism reflects systemic changes caused by inflammatory responses and oxidative stress (35). Lysophatidylcholine (lysoPCs) is positively correlated with insulin sensitivity and pro-inflammatory properties (36). Plasma metabolomics analysis showed that lysoPCs and lysoPEs were correlated with OGTT AUCglucose and HOMA-IR, and their plasma levels significantly reduced in patients with T2DM (37). In the blood, increased levels of LPC and PC indicate that an oxidative stress response is aroused (38). Lecithin (PE) is decarboxylated in the body by serine to produce choline, which improves the speed and accuracy of information transmission between different nerve cells. Sphingolipid metabolites are key signaling molecules involved in immunity and inflammation and many cellular regulatory processes (35). In this study, it was found that dietary intervention with MBPs inhibited oxidative stress injury and lipid peroxidation by regulating glycerophospholipid and sphingolipid metabolism, thus to alleviate IR in HFD mice.

Dyslipidaemia and increased IR can be caused by lipid metabolism disorders, particularly lipids with low carbon and low double bond numbers (39). In this study, the metabolic spectrum of mice showed that the contents of trans oleic acid and pentadecanoic acid in serum decreased after MBPs dietary intervention compared with HFD model group. Trans-oleic acid can induce impaired insulin receptor signaling and fat accumulation in mature adipocytes (40). Oleic acid is a potential biomarker for liver injury, such as obesity (41). Free fatty acids promote oxidative stress and inflammation by accumulating lipid derivatives and destroying mitochondrial function to reduce the insulin hypoglycaemic effect (39). 1, 11-Undecanedicarboxylic acid and phytic acid may indicate peroxisome disorders, Peroxisome proliferator-activated receptor γ (PPARγ) has been shown to regulate lipid and glucose homeostasis, cell differentiation, and growth regulation (42). In this study, after MBPs dietary intervention, 1,11-undedicarboxylic acid in mouse serum decreased significantly, indicating that 1,11-undedicarboxylic acid plays a role in regulating insulin resistance by regulating lipid metabolism and glucose homeostasis.

Nicotinamide (NAM), an important metabolite of nicotinic acid and nicotinamide metabolism, can be converted from nicotinamide ribosomes (NR) to NMN, which is the precursor of nicotinamide adenine dinucleotide (NAD^+^). NAD^+^ regulates the citric acid cycle, cytoplasmic glycolysis, gluconeogenesis, glycogen metabolism, and mitochondrial fatty acid oxidation (43). An imbalance in NAD^+^ homeostasis is associated with mitochondrial dysfunction, IR, and obesity. In HFD mice, NAD^+^ biosynthesis dependent on niacinamide phosphate glycosyltransferase (NAMPT) is impaired in metabolic organs, which leads to severe IR. NAD^+^ and its metabolites play important roles in insulin sensitivity and glucose tolerance in rodents. Supplementation of NAD precursors has been shown to prevent and reverse IR, mitochondrial dysfunction, and liver damage in HFD-induced obesity mouse models (44).

Trigonelline in alkaloid metabolism can significantly reduce the production of reactive oxygen species (ROS), the concentration of superoxide dismutase (SOD), and glutathione peroxidase (GPx), and considerably reduce the increased levels of TNF-α and IL-6, it also induces the activation of the PI3K/Akt pathway (45). This is consistent with the results of this study. After MBPs dietary intervention, the down-regulation of choline level in liver tissue may also indicate the recovery of cell membrane integrity after MBPs dietary intervention. The remission of insulin resistance in mice may be related to the activation of the PI3K/Akt signaling pathway, which will be further studied in the future. The results of this study provide theoretical support for the possible application of MBPs in the adjuvant treatment of metabolic diseases such as obesity, T2DM, and non-alcoholic fatty liver because of IR and the design and production of hypoglycaemic functional foods.

## Conclusion

This experiment was conducted to study the alleviating effect of the MBPs dietary intervention on HFD-induced IR in mice. In this study, body weight, glucose homeostasis and islet function of HFD mice were improved, systemic inflammation and oxidative stress response were controlled, and insulin resistance symptoms were relieved. Serum metabolomics revealed the changes of serum metabolites and metabolic pathways after MBPs dietary intervention. One biomarker associated with IR is glycine. In addition, the four important differential metabolites, pyroglutamate, D-glutamine, adipic acid, and nicotinamide, were involved in changes in 12 metabolic pathways. MBPs dietary intervention may play a positive role in regulating metabolic pathways such as amino acids, fatty acids, glycerophospholipids, and alkaloids.

## Data Availability Statement

The raw data supporting the conclusions of this article will be made available by the authors, without undue reservation.

## Ethics Statement

The animal study was reviewed and approved by Animal Experiment Committee of Heilongjiang Bayi Agricultural University.

## Author Contributions

LL: writing—original draft, methodology, data curation, review, and editing. YT: methodology, data curation, review, and editing. YF and SZ: analysis of metabolomics results and proof reading. YJ and YiZ: methodology and data analysis. YuZ: information analysis and literature guarantee. CW: conceptualization, project administration, resources, funding acquisition, and supervision. All authors have made substantial contributions to conception and design of the project. All authors have critically revised and approved the final submitted version of the manuscript.

## Funding

This work was supported by National Key Research and Development Programs (Grant No. 2018YFE0206300), Heilongjiang Province Engineering Science and Technology Major Special Project of China (Grant No. 2021ZX12B06), and Graduate Innovation Project of Heilongjiang Bayi Agricultural University (Grant No. YJSCX2021-Z04).

## Conflict of Interest

The authors declare that the research was conducted in the absence of any commercial or financial relationships that could be construed as a potential conflict of interest.

## Publisher's Note

All claims expressed in this article are solely those of the authors and do not necessarily represent those of their affiliated organizations, or those of the publisher, the editors and the reviewers. Any product that may be evaluated in this article, or claim that may be made by its manufacturer, is not guaranteed or endorsed by the publisher.
